# KLF3 is a crucial regulator of metastasis by controlling STAT3 expression in lung cancer

**DOI:** 10.1002/mc.23072

**Published:** 2019-09-05

**Authors:** Wei Sun, Shan Hu, Yukun Zu, Yu Deng

**Affiliations:** ^1^ Department of Thoracic Surgery, Tongji Hospital Huazhong University of Science and Technology Wuhan China

**Keywords:** KLF3, lung cancer, metastasis, STAT3

## Abstract

Lung cancer is one of the most common causes of cancer‐related mortality worldwide, which is partially due to its metastasis. However, the mechanism underlying its metastasis remains elusive. In this study, we showed that a low Krüppel‐like factor 3 (KLF3) expression level is correlated with a poor prognosis and TNM stages in clinical patients with lung cancer and further demonstrated that KLF3 expression is downregulated in lung cancer tissues compared with adjacent normal samples. In addition, bioinformatics analysis results showed that KLF3 expression is related to lung cancer epithelial‐mesenchymal transition (EMT). In vitro and in vivo experiments also showed that KLF3 silencing promotes lung cancer EMT and enhances lung cancer metastasis. More importantly, bioinformatics analysis and in vitro experiments indicated that the role of KLF3 in lung cancer metastasis is dependent on the STAT3 signaling pathway. Overall, our data indicated the crucial function of KLF3 in lung cancer metastasis and suggested opportunities to improve the therapy of patients with lung cancer.

## 1 INTRODUCTION

Lung cancer is one of the most common causes of cancer‐related mortality worldwide.^1^, ^2^ To date, various clinical advances in lung cancer therapies, including surgical resection and chemotherapy, have led to certain achievements.^3^, ^4^ However, the prognosis of advanced lung cancer is very poor, mainly due to cancer metastasis. Many efforts have been made to explore the molecular mechanism of tumor metastasis. However, the precise mechanism of tumor metastasis remains elusive.^5^ Thus, to identify novel therapeutic targets and improve the 5‐year survival rate of patients with lung cancer, there is an urgent need to attain an improved understanding of the complicated molecular mechanisms of lung cancer metastasis.

Krüppel‐like factors (KLFs) contain homologous zinc finger structures that bind to GC‐rich regions in DNA.^6^ Increasing research has shown that KLFs are involved in biological processes by regulating gene expression at the transcriptional level.^7^ The aberrant expression of KLFs has been correlated with tumor biology. For example, the aberrant expression of KLF4 regulates tumor development by regulating the Notch1 pathway.^8^ Some studies have shown that KLF4 suppresses the proliferation of tumors, including breast, and liver cancer tumors.^9^, ^10^ In contrast, knockdown of KLF4 inhibits breast cancer metastasis mediated by the Notch pathway.^11^ These results indicated that KLF4 plays multiple roles in tumor development, including tumor initiation and metastasis. In addition, the roles of other KLFs in cancer metastasis have also been extensively explored in different types of tumors.^12^, ^13^, ^14^, ^15^ KLF3, which is a member of the Krüppel‐like factor family, has also shown aberrant expression in many kinds of cancers. In human metastatic sarcomas, dysregulated expression of KLF3 has been observed, and the knockdown of KLF3 has been shown to promote cancer cell metastasis.^16^ RNA sequencing analysis has demonstrated that KLF3 may play a protective role in colorectal cancer.^17^ However, the precise mechanism of the regulation of cancer metastasis mediated by KLF3 remains incompletely explained, and the role of KLF3 in lung cancer remains unclear.

Metastasis is a multistep process that is dependent on the regulation of abnormal signal transduction through pathways, including the TGFβ, JAK2/STAT3, and Wnt signaling pathways.^18^ Signal transducer and activator of transcription 3 (STAT3) is a member of the STAT family, which is composed of STAT1, STAT2, STAT3, STAT4, STAT5A, STAT5B, and STAT6.^19^, ^20^ Under normal conditions, STAT3 mainly localizes in the cytoplasm, and upon stimulation from external environmental factors, such as IL6, STAT3 translocates into the nucleus and binds to DNA to activate the expression of related genes. In multiple cell types, increasing evidence has demonstrated that STAT3 functions as a tumor oncogene. Increasing studies have shown that the overexpression of STAT3 enhances cell proliferation and metastasis in a variety of cancers. For example, the overexpression of IL‐22RA1 promotes stemness and tumorigenicity in pancreatic cancer by regulating the STAT3 signaling pathway.^21^ In addition, it has recently been shown that the activation of the STAT3 signaling pathway promotes colon cancer metastasis by regulating epithelial‐mesenchymal transition (EMT).^22^, ^23^ The STAT3 signaling pathway seems to be necessary for cancer metastasis. Despite these findings, the reason for the abnormal activation of STAT3, especially in lung cancer, has yet to be determined.

Considering the urgent need to explore novel therapeutic targets for lung cancer metastasis, we determined the potential role of KLF3 in lung cancer metastasis and examined the expression levels of KLF3 in clinical lung cancer tissues in this study. More importantly, we found that the biological function of KLF3 in metastasis is dependent on transcriptional STAT3 expression. Our findings suggested that the KLF3/STAT3 signaling pathway is a potential therapeutic target for patients with lung cancer.

## 2 MATERIALS AND METHODS

### 2.1 Reagents and cell culture

Lipofectamine 2000 reagent was obtained from Invitrogen (Shanghai, China). Matrigel was purchased from BD Biosciences (Becton, Dickinson and Company). A549 and H1299 cells were purchased from the American Type Culture Collection and were cultured in Dulbecco's modified Eagle's medium (DMEM) supplemented with 10% fetal bovine serum (FBS) at 37°C in a humidified incubator with 5% CO_2_. siRNA‐STAT3 (small interfering RNA) and negative control siRNA (siNC) were purchased from RiboBio (Guangzhou, China). Antibodies against STAT3, KLF3, E‐cadherin, and N‐cadherin were obtained from Cell Signaling Technology (Boston), and antibodies against vimentin, Zo‐1, and glyceraldehyde‐3‐phosphate dehydrogenase (GAPDH) were purchased from Abcam.

### 2.2 Western blot analysis

Western blot analysis was performed using total cell lysates that were lysed in NP40 (150 mM NaCl, 0.1% SDS, 1% NaMoO_4_, 1% NP‐40, 50 mM Tris‐HCl [pH 7.5], and 0.02% NaN_3_) buffer with protease and phosphatase inhibitors (Roche). The protein samples were separated by sodium dodecyl sulfate polyacrylamide gel electrophoresis and transferred onto polyvinylidene difluoride (PVDF) membranes (Millipore). The bands were incubated with primary antibodies at 4°C overnight. Next, the bands were incubated with HRP‐conjugated secondary antibodies. Then, signals were examined by ECL reagents (Thermo Fisher Scientific).

### 2.3 RNA isolation and quantitative real‐time polymerase chain reaction

Total RNA was extracted from cells with TRIzol reagent (Invitrogen, Carlsbad, CA). Then, complementary DNA (cDNA) was synthesized with a PrimeScript RT reagent kit (Takara, Dalian, China). Quantitative real‐time polymerase chain reaction (qRT‐PCR) was performed with SYBR Green Master Mix (Takara, Dalian, China). The expression of the indicated genes was normalized to the expression of β‐actin. The relative expression was determined using the $2^{-\Delta \Delta C_{t}}$ method. The qRT‐PCR assays were performed with the following primers: GAPDH gene:

5′‐GGAGCGAGATCCCTCCAAAAT‐3′ (forward),

5′‐GGCTGTTGTCATACTTCTCATGG‐3′ (reverse);

KLF3 gene:

5′‐TGTCTCAGTGTCATACCCATCT‐3′ (forward),

5′‐CCTTCTGGGGTCTGAAAGAACTT‐3′ (reverse);

E‐cadherin gene:

5′‐CGAGAGCTACACGTTCACGG‐3′ (forward),

5′‐GGGTGTCGAGGGAAAAATAGG‐3′ (reverse);

vimentin gene:

5′‐GACGCCATCAACACCGAGTT‐3′ (forward),

5′‐CTTTGTCGTTGGTTAGCTGGT‐3′ (reverse);

ZO‐1 gene:

5′‐CAACATACAGTGACGCTTCACA‐3′ (forward),

5′‐CACTATTGACGTTTCCCCACTC‐3′ (reverse);

N‐cadherin gene:

5′‐TCAGGCGTCTGTAGAGGCTT ‐3′ (forward),

5′‐ATGCACATCCTTCGATAAGACTG ‐3′ (reverse);

and STAT3 gene:

5′‐CAGCAGCTTGACACACGGTA ‐3′ (forward),

5′‐AAACACCAAAGTGGCATGTGA ‐3′ (reverse).

### 2.4 Plasmid constructs and stable cell lines

The short hairpin RNA (shRNA) sequences for KLF3 were obtained from Sigma‐Aldrich. shKLF3 that was used in all the experiments was constructed using the following sequence: CCGGCCCACTTGAAAGCACACAGAACTCGAGTTCTGTGTGCTTTCAAGTGGGTTTTT, in a pLKO.1‐puromycin vector. A scrambled vector was used as a control (nontargeted control). For lentiviral production, PLKO.1‐shRNAKLF3 packaging vector (pCMV‐dr8.Z dvpr) and envelope (pCMV‐VSV‐G) plasmids were cotransfected into 293T cells using Lipofectamine 2000 (Invitrogen). After 72 hours, lung cancer cells were infected with viral particles. Stable cell lines were selected by culturing cells in medium with 2 μg/mL puromycin for 2 weeks. For STAT3‐binding luciferase reporter constructs, we amplified the promoter of the KLF3 gene containing the predicted binding site for STAT3. Next, the promoter of the KLF3 gene fragment was inserted into pGL3 (Promega). For the mutated KLF3 promoter luciferase reporter constructs, we first mutated the binding site for STAT3, and the mutated promoter gene fragment was then cloned into pGL3. The following KLF3 promoter fragment primers were used: 5′‐CATTTAGCCAAGAGGAATTTGGCG‐3′ (forward) and 5′‐TCCCAGTCTGCGCCGCCGCAGCT‐3′ (reverse).

### 2.5 Luciferase reporter assay

For the luciferase reporter assay, cells were seeded into 24‐well plates. The cells were cotransfected with WT‐STAT3 promoter‐PGL3 and mutated STAT3 promoter‐PGL3 luciferase constructs and a Renilla plasmid into the indicated cells overnight. Then, luciferase activity was analyzed with a Dual‐Luciferase Reporter Assay System after 24 hours (Promega, CA) according to the manufacturer's instructions.

### 2.6 Clinical samples and immunohistochemical staining

Human lung cancer specimens and adjacent normal samples were obtained from Tongji Hospital, Huazhong University of Science and Technology. The expression of KLF3, E‐cadherin and vimentin in lung cancer tissues that were collected between 2015 and 2018 was examined using immunohistochemistry (IHC). Informed consent was obtained from each patient. This study was conducted with approval by the Huazhong University of Science and Technology Institute Research Ethics Committee. All specimens were quickly stored in liquid nitrogen. The expression levels of KLF3, E‐cadherin, and vimentin were scored as the proportion of the stained area (0%, 0; 1%‐25%, 1; 25%‐50%, 2; 50%‐75%, 3; and 75%‐100%, 4) multiplied by the staining intensity (0, *negative*; 1, *weak*; 2, *moderate*; and 3, *high*).

### 2.7 Wound‐healing and transwell assays

Cells were plated into six‐well plates at a confluence of 90%. After 24 hours, wounds were made with a 200 μL pipette tip. Then, the cells were cultured in serum‐free medium. Wound healing distances were observed at different timepoints. For the Transwell migration assay, cells were plated into an upper chamber of a Transwell and cultured with serum‐free medium. The lower chamber was filled with complete medium supplemented with 10% FBS. After incubation for 24 hours, the inserts were fixed with 4% methanol and stained with 0.1% crystal violet. For the Transwell invasion assay, cells were seeded into the upper chamber of a Transwell that was pretreated with Matrigel (BD Biosciences, Sparks, MD), and the lower chamber was filled with complete medium supplemented with 10% FBS. Images were acquired by a microscope. At least three independent experiments were performed with triplicate samples for each experiment.

### 2.8 Chromatin immunoprecipitation assay

Cells were plated into dishes and treated according to the requirements of the experiment. The chromatin immunoprecipitation (ChIP) assay was performed using a ChIP Assay Kit (Thermo Fisher Scientific). Cells were fixed with 1% formaldehyde and then examined by the ChIP assay according to the manufacturer's instructions. ChIP‐DNA was used for PCR. Primers were designed to examine the promoter region of STAT3 from 1745 to 2020. ChIP PCR was performed with the following primers: 5′‐GATGGAACGGAGTACGGGGTT‐3′ (forward) and 5′‐ATCAGCTAGTTAGATAGTC‐3′ (reverse) for the human STAT3 gene promoter; and 5′‐TACTAGCGGTTTTACGGGCG‐3′ (forward) and 5′‐TCGAACAGGAGGAGCAGAGAGCGA‐3′ (reverse) for the human *GAPDH* promoter.

### 2.9 Animal experiments

Four‐week‐old male BALB/c nude mice were obtained from the Shanghai SLRC Laboratory Animal Co, Ltd. A total of 2 × 10^6^ lung cancer cells were injected into the nude mice through the lateral vein. After 1 week, tumor sizes were measured every 5 days. The mice were killed, and metastatic lung tissues were collected and weighed after 4 weeks. The animal experiments were approved by the Institutional Animal Care and Use Committee of Huazhong University of Science and Technology.

### 2.10 Statistical analysis

The data are presented as the mean ± standard deviation (SD). Pearson's *χ* ^2^ test was used to analyze associations between KLF3, E‐cadherin, and vimentin to evaluate relationships between KLF3 expression and clinicopathological characteristics. *P* < .05 was considered statistically significant. All statistical analyses were performed using SPSS 21.0 statistical software (SPSS Inc, Chicago, IL) and GraphPad Prism 5.0 software (GraphPad Software, Inc, La Jolla, CA).

## 3 RESULTS

### 3.1 Reduced KLF3 expression in human lung cancer is associated with tumor progression

**Figure 1:**
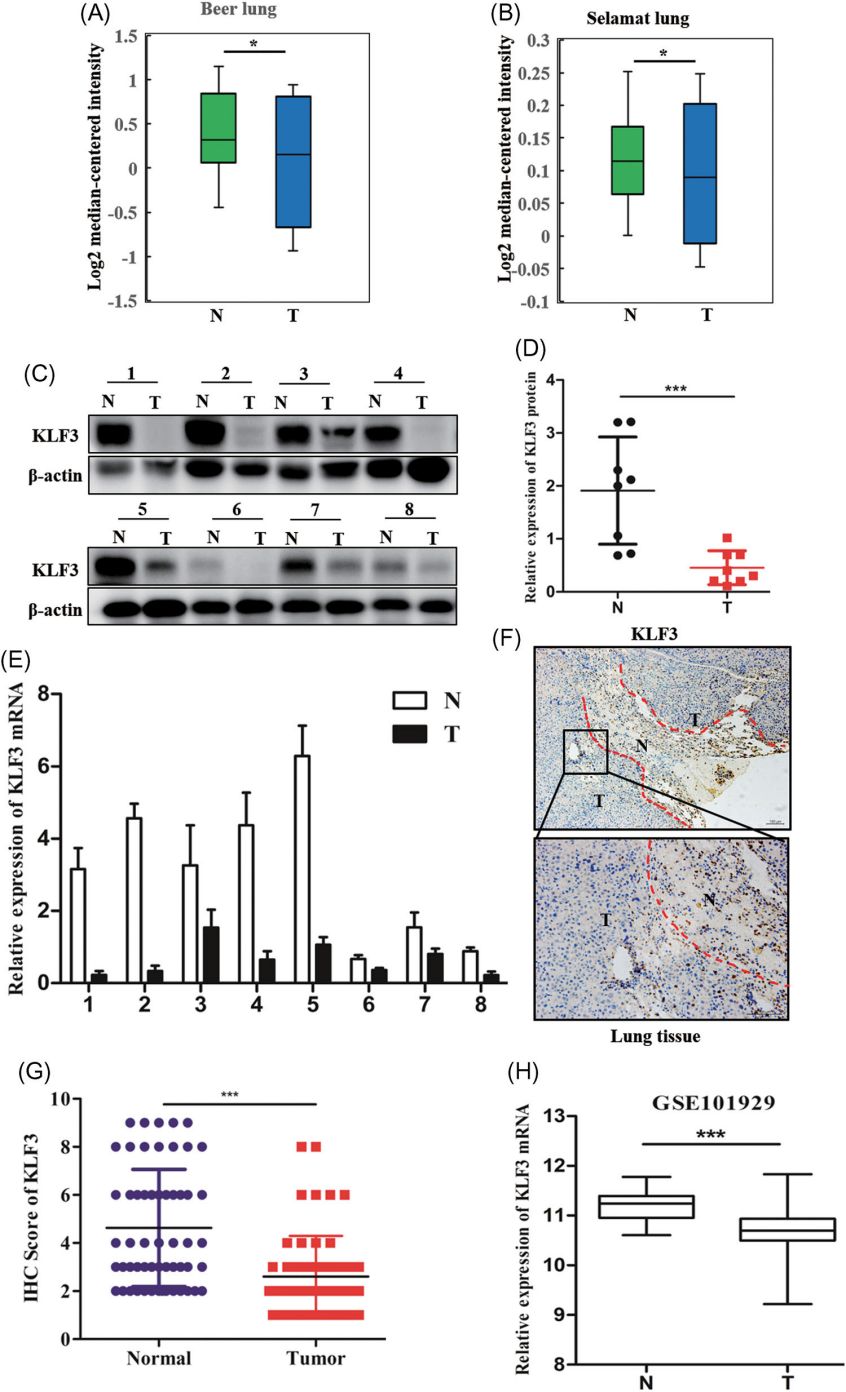
Reduced KLF3 expression in human lung cancer tissues is associated with tumor progression. A and B, Analysis of KLF3 mRNA expression in lung cancer tissues (T) and normal samples (N). The data were obtained from the Oncomine database (*P < .05). C and D, The levels of KLF3 protein expression were examined in eight pairs of lung cancer tissues and normal samples. The quantification of KLF3 is shown in the diagram (***P < .001). E, Analysis of KLF3 protein expression by IHC in 89 paired lung cancer tissues and normal specimens. The images show a representative lung cancer tumor with adjacent normal tissues. F, Quantification of KLF3 expression using the IHC results is shown in the diagram. G, Analysis of KLF3 mRNA expression was conducted on eight pairs of lung cancer tissues and normal samples (***P < .001). H, The expression levels of KLF3 mRNA in the GEO database (GSE101929). IHC, immunohistochemistry; KLF3, Krüppel‐like factor 3; mRNA, messenger RNA; N, normal; T, tumor [Color figure can be viewed at wileyonlinelibrary.com]

To investigate the role of KLF3 in human lung cancer progression, we first examined KLF3 expression in normal and cancerous human lung tissues by bioinformatics analysis (data were obtained from the Oncomine database). As shown in Figure 1A and 1B, we found that the messenger RNA (mRNA) expression of KLF3 was markedly downregulated in lung cancer tissues compared to normal tissues. Next, as shown in Figure 1C and 1D, we found that the protein expression of KLF3 was markedly downregulated in eight matched lung cancer and normal tissues. Furthermore, we examined the mRNA expression levels of KLF3 in the abovementioned tissues, and reduced KLF3 mRNA expression was observed in the lung cancer specimens (Figure 1E). In addition, the levels of KLF3 expression were further evaluated by IHC. Similar to the abovementioned results, we observed that the levels of KLF3 expression were reduced in lung cancer specimens compared with normal tissues (Figure 1F and 1G).

To further assess the clinical value of KLF3, we first detected the KLF3 protein expression levels in 56 pairs of human lung cancer tissues and matched normal lung tissues. As shown in Table 1, we found that the levels of KLF3 expression were negatively correlated with the tumor, node, metastasis (TNM) stage and lymph node metastasis. Importantly, the bioinformatics analysis results also showed that KLF3 expression was positively correlated with the TNM stage (Figure 1H). Collectively, the abovementioned data indicated that KLF3 expression is downregulated in lung cancer tissues and closely related to tumor progression and might act as a molecular marker for predicting lung cancer metastasis.

**Table 1:** Association between clinicopathological characteristics and expression of KLF3 in lung cancer patients (n = 56)

Characteristics	n	KLF3		*P* value
		High expression	Low expression	
Sex				
Male	26	12	14	.789
Female	30	16	14	
Age				
<60	32	15	17	.43
≥60	24	14	10	
Differential grade				
Poor	14	6	8	.587
Middle	17	9	8	
Well	25	15	10	
LN metastasis				
Positive	23	9	14	.03
Negative	33	23	10	
TNM stage				
I‐II	29	18	11	.037
III‐IV	27	9	18	

Abbreviations: LN, lymph node; TNM, tumor, node, metastasis.

### 3.2 Knockdown of KLF3 promotes lung cancer cell migration and invasion

**Figure 2:**
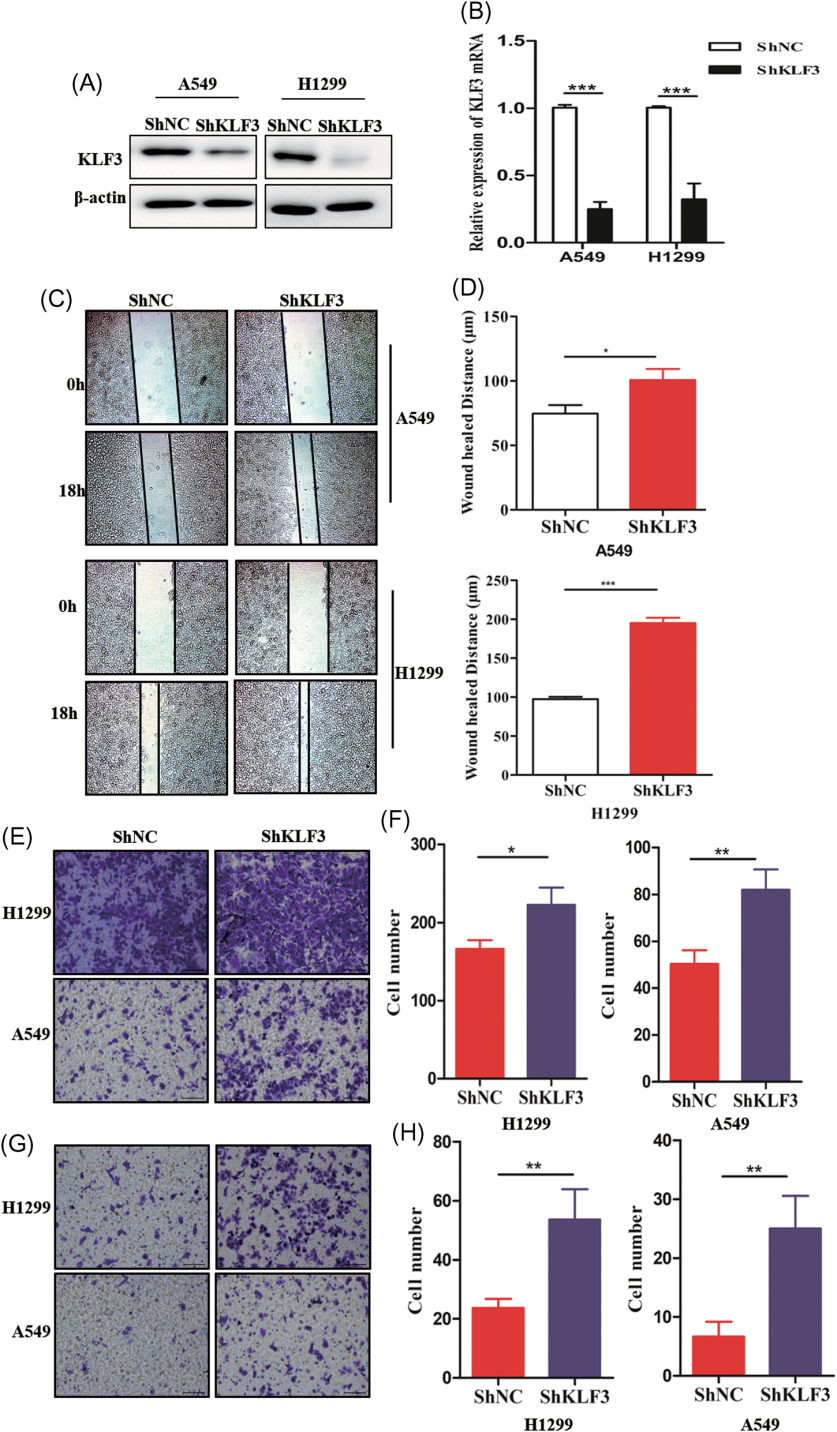
Knockdown of KLF3 promotes lung cancer cell migration and invasion. A, Western blot analysis results showing KLF3 protein expression levels in H1299 and A549 cells expressing shKLF3. B, qPCR results showing KLF3 mRNA expression levels in H1299 and A549 cells expressing shKLF3 (***P < .001). C, The wound‐healing assay showing the effects of KLF3 knockdown on the migratory ability of H1299 and A549 cells. D, The quantification of the migratory distance is shown in the histogram (*P < .05 and ***P < .001). E, The Transwell‐migration assay showing the effects of KLF3 silencing on the migratory ability of H1299 and A549 cells. F, The quantification of the migratory ability is shown in the histogram (*P < .05 and **P < .01). G, The Transwell‐invasion assay showing the effects of KLF3 knockdown on the invasion ability of H1299 and A549 cells. H, The quantification of the invasion ability is shown in the histogram (**P < .01). KLF3, Krüppel‐like factor 3; NC, negative control; qPCR, quantitative real‐time polymerase chain reaction [Color figure can be viewed at wileyonlinelibrary.com]

**Figure 3:**
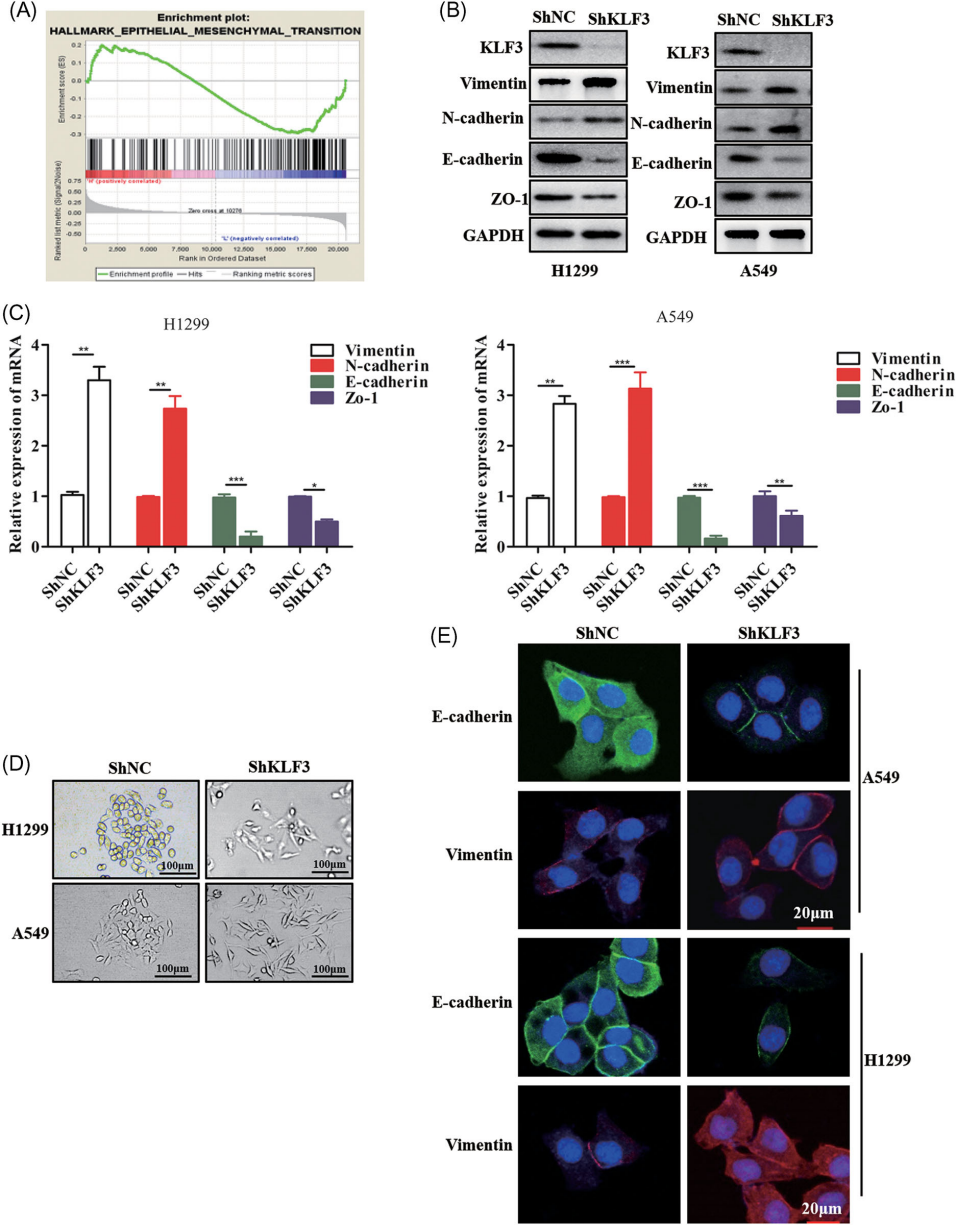
KLF3 silencing promotes EMT in lung cancer. A, Gene set enrichment analysis (GSEA) results showing the expression of EMT‐related genes between patients with high KLF3 expression levels and patients with low KLF3 expression level. The data were obtained from the GEO101929 database. B, Western blot showing the expression of EMT‐related proteins in shKLF3 cells. C, qPCR results showing the mRNA expression of EMT‐related genes in shKLF3 cells (*P < .05, **P < .01, and ***P < .001). D, Representative images of H1299 and A549 cells showing epithelial and mesenchymal features. The scale bar = 100 μm. E, Representative immunofluorescence staining images of EMT‐related proteins in shNC and shKLF3 cells. The scale bar = 20 μm. EMT, epithelial‐mesenchymal transition; GAPDH, glyceraldehyde‐3‐phosphate dehydrogenase; KLF3, Krüppel‐like factor 3; NC, negative control; qPCR, quantitative real‐time polymerase chain reaction [Color figure can be viewed at wileyonlinelibrary.com]

Considering that KLF3 is related to lung cancer metastasis, we established stable cell lines expressing shRNA‐KLF3 via lentiviral infection. As shown in Figure 2A and 2B, the silencing efficiency was analyzed by Western blot and qPCR assays in H1299 and A549 cells. Then, we examined the migratory ability of control and shRNA‐KLF3 cells. As shown in Figure 2C and 2D, the wound‐healing assay surprisingly showed that the knockdown of KLF3 markedly promoted cell migration. Consistent with these results, the Transwell assay using Transwells coated with and without Matrigel showed that the knockdown of KLF3 substantially promoted the migration (Figure 3E and 3F) and invasion (Figure 3G and 3H) of lung cancer cells.

To assess the role of KLF3 in lung cancer cell proliferation, we compared changes in cell proliferation between the three groups. As shown in Figure S1, no significant changes were observed between the three groups, indicating that the promotion of migration and invasion by KLF3 knockdown was not dependent on cell proliferation. Overall, the abovementioned data further implicated that the knockdown of KLF3 enhances lung cancer migration and invasion and that KLF3 might suppress the metastasis of lung cancer cells.

### 3.3 KLF3 silencing promotes EMT in lung cancer

Metastasis is a multistep process. EMT is considered an important step in the metastasis cascade and enables cell migration and invasion. Indeed, the enrichment of genes that mediate EMT in lung cancer tissues with low KLF3 expression levels was observed (Figure 3A). This finding led us to hypothesize that KLF3 regulates the EMT of cancer cells that affects metastasis. To evaluate the effect of KLF3 on EMT, we first examined the protein expression of mesenchymal and epithelial markers in control and shRNA‐KLF3 cells. Interestingly, the expression levels of the mesenchymal markers were high in the shRNA‐KLF3 cells compared to the control cells (Figure 3B and 3C). More importantly, shKLF3 cells exhibited an obvious mesenchymal transition phenotype (Figure 3D). The immunofluorescence (IF) assay results also showed that the shRNA‐KLF3 cells expressed substantially more mesenchymal markers than the control cells (Figure 3E). Consistent with the phenomena that are affected by KLF3 knockdown, compared with the control cells, the shRNA‐KLF3 cells obviously exhibited a mesenchymal phenotype throughout the general observation period.

Collectively, these data suggest that KLF3 silencing mediates EMT progression and that KLF3 is involved in lung cancer development.

### 3.4 KLF3 silencing upregulates STAT3 expression in a transcriptional manner

**Figure 4:**
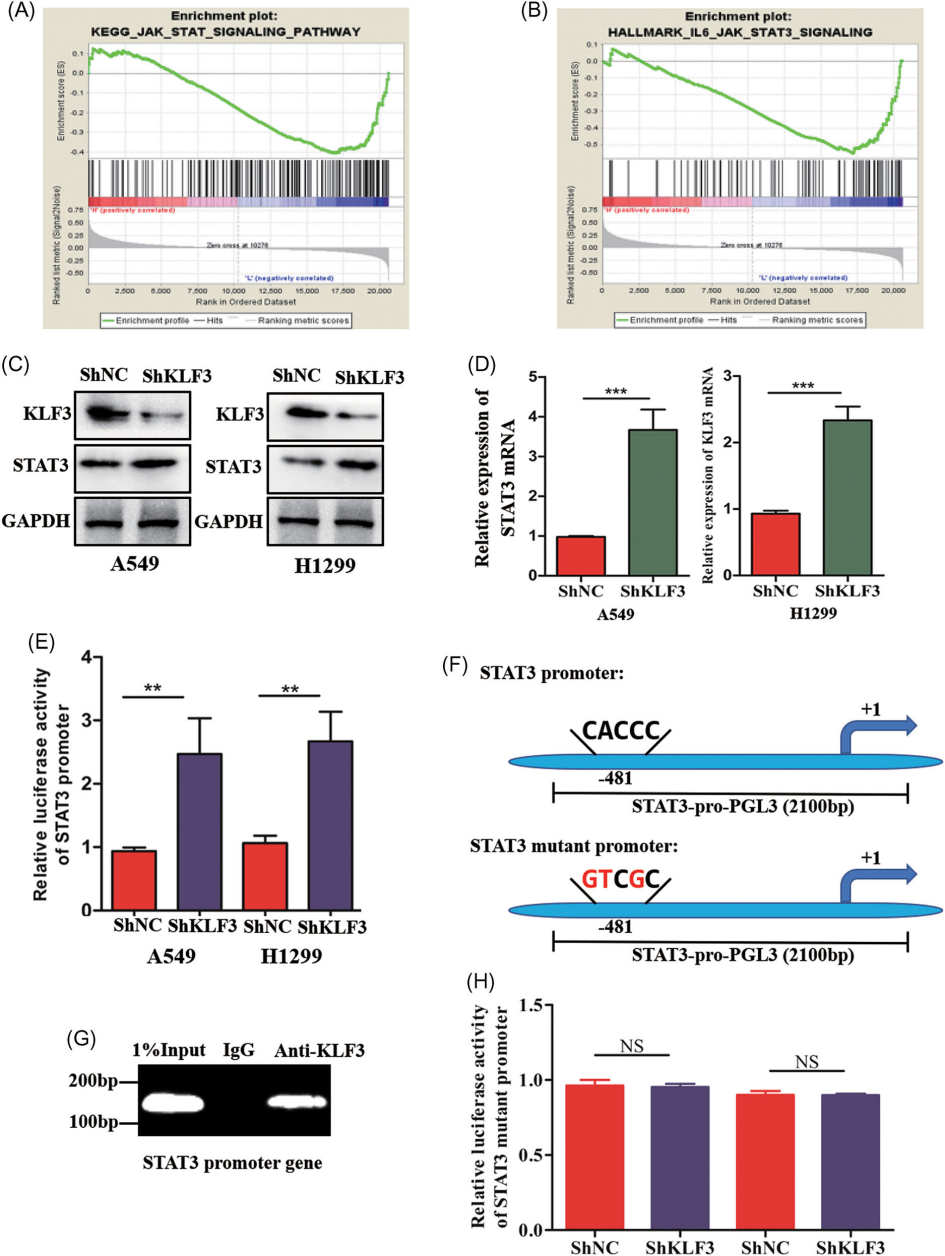
KLF3 silencing upregulates STAT3 expression in a transcriptional manner. A and B, KEGG and Hallmark analysis showing that the enrichment of STAT3 target gene sets is associated with low KLF3 expression levels in lung cancer tissues. C, Western blot analysis showing the protein expression of STAT3 in shNC and shKLF3 cells. D, qPCR results showing the mRNA expression of STAT3 in shNC and shKLF3 cells (***P < .001). E, Relative luciferase activities were examined in shNC and shKLF3 cells (**P < .01). F, A schematic of the STAT3 promoter luciferase construct that was predicted with the locations of the KLF3 binding site and the mutated sequences of the KLF3 binding site (bottom). G, KLF3 binding on the promoter site of STAT3 was evaluated by a CHIP assay in H1299 cells. H, Relative luciferase activities were examined in shNC and shKLF3 cells transfected with the indicated plasmids. KLF3, Krüppel‐like factor 3; STAT3, signal transducer and activator of transcription 3; mRNA, messenger RNA; NC, negative control; NS, not significant [Color figure can be viewed at wileyonlinelibrary.com]

To explore the molecular mechanism underlying how KLF3 modulates metastasis in lung cancer cells, we further analyzed an online database by gene set enrichment analysis (GSEA). Surprisingly, we found that the enrichment of STAT3 target gene sets is associated with reduced KLF3 expression in lung cancer (Figure 4A and 4B), suggesting that KLF3 may be involved in the STAT3 signaling pathway. We determined whether KLF3 regulates STAT3 expression in lung cancer cells. As shown in Figure 4C, the Western blot results showed that the STAT3 expression level was reduced in control cells compared with shKLF3 cells. It has been reported that KLF3 mainly controls gene expression by epigenetically regulating gene transcription. Therefore, the mRNA expression levels of STAT3 were examined in control cells and shKLF3 cells. Using qPCR assays, we observed that the knockdown of KLF3 markedly increased the STAT3 mRNA expression level (Figure 4D). Moreover, to confirm the notion that KLF3 regulates STAT3 expression in a transcriptional manner, we constructed a luciferase plasmid containing the promoter of the STAT3 gene. Next, we cotransfected luciferase plasmids into cells. The dual‐luciferase assay results demonstrated that compared with control cells, the knockdown of KLF3 strongly increased the luciferase activity of theSTAT3 promoter (Figure 4E).

Considering the role of KLF3 in regulating gene expression, it is dependent on the *CACCC* box‐binding transcription element. Thus, we speculated that KLF3 directly interacts with the promoter of STAT3 and subsequently decreases its expression level. As expected, we predicted KLF3 binding to the STAT3 promoter region (Figure 4F). To confirm this speculation, Chip assays were performed using an anti‐KLF3 antibody to pull‐down KLF3‐bound chromatin and PCR using primers for the STAT3 promoter. The ChIP results clearly suggested that KLF3 binds to the STAT3 gene promoter (Figure 4G), indicating that KLF3 might suppress STAT3 expression by binding to the promoter of the STAT3 gene. In addition, we further mutated the binding site of the STAT3 promoter that is specifically recognized by KLF3 (Figure 4F). Importantly, the dual‐luciferase assays showed that the knockdown of KLF3 had no effect on the luciferase activity of the mutated STAT3 promoter (Figure 4H). Overall, our data suggested that KLF3 regulates STAT3 expression in a transcriptional manner.

### 3.5 KLF3 silencing promotes metastasis through the upregulation of STAT3 expression

**Figure 5:**
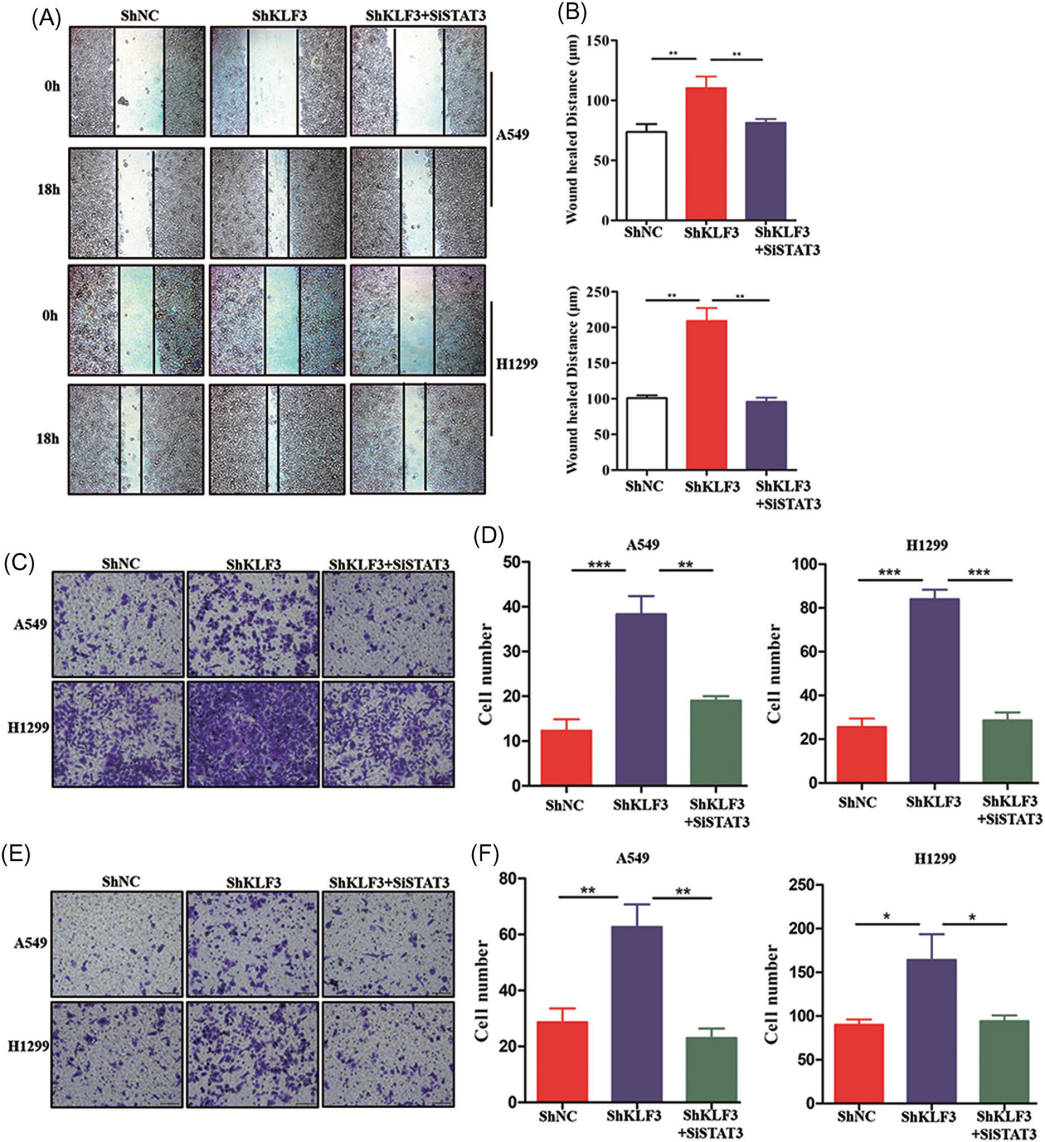
KLF3 silencing promotes metastasis through the upregulation of STAT3 expression. A, Wound‐healing assays were performed in shKLF3 cells with or without siSTAT3. Representative images are shown. B, The quantification of the wound healing distance is shown in the histogram (**P < .01). C, Transwell‐migration assays were performed in shKLF3 cells with or without siSTAT3. Representative images are shown. D, The quantification of the migratory ability is shown in the histogram (**P < .01 and ***P < .001). E, Transwell invasion assays were performed in shKLF3 cells with or without siSTAT3. Representative images are shown. F, The quantification of the invasion ability is shown in the histogram (*P < .05 and **P < .01). KLF3, Krüppel‐like factor 3; NC, negative control [Color figure can be viewed at wileyonlinelibrary.com]

Considering the crucial role of STAT3 in tumor progression and the contribution of KLF3 to the effects of STAT3, we examined whether STAT3 is essential for metastasis‐mediated knockdown of KLF3 in lung cancer cells. First, we transfected shRNAs to knockdown STAT3 expression in shKLF3 cells. As shown in Figure 5A and 5B, the wound healing assay showed that STAT3 silencing markedly abrogated the knockdown of KLF3‐induced cell migration. Furthermore, the Transwell assays showed that the knockdown of STAT3 abolished the migration‐ and invasion‐promoting effects induced by the knockdown of KLF3 (Figure 5C‐F). These data indicated that STAT3 expression is at least partially essential for the prometastatic function of reducing the KLF3 expression level in lung cancer cells.

### 3.6 Knockdown of KLF3 promotes lung cancer metastasis in vivo

**Figure 6:**
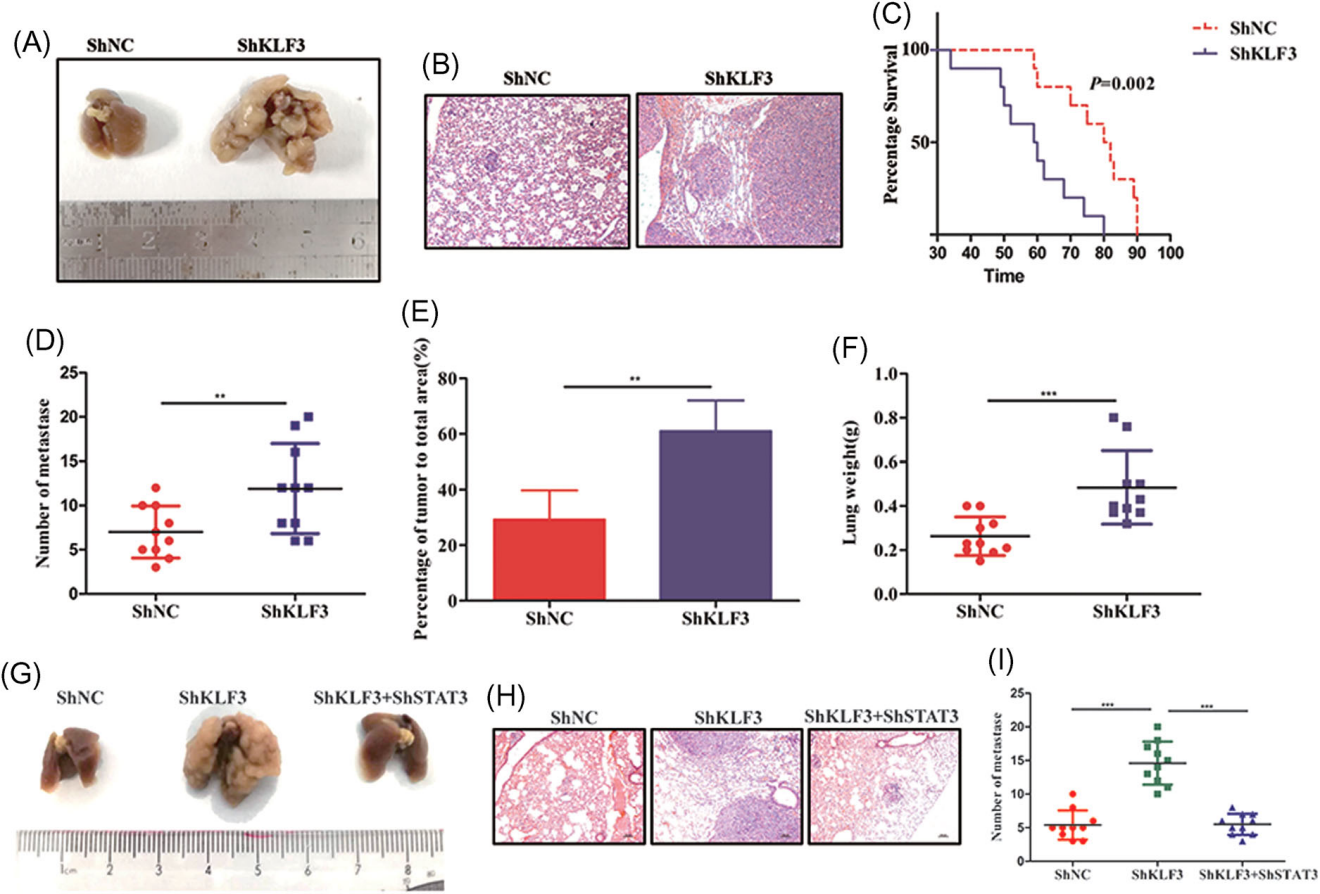
Knockdown of KLF3 promotes lung cancer metastasis in vivo. A, Representative images of lung tissues with metastatic nodules in the two groups are shown. B, Representative HE images of the metastatic lung nodules in the two groups are shown. C, Kaplan‐Meier survival analysis showing the overall survival of mice administered shNC or shKLF3 A549 cells. D, A scatter plot showing metastatic lung nodules on the surface of the lungs (**P < .01). E and F, A scatter plot showing the wet weight of the lungs in the two groups and the percentage of metastatic nodules to the total lung area (**P < .01 and ***P < .001). G, Representative images of lung tissues with metastatic nodules are shown for the three groups. H, Representative HE images of metastatic lung nodules from the three groups are shown. I, A scatter plot showing metastatic lung nodules on the surface of the lungs in the three groups. ***P < .001. KLF3, Krüppel‐like factor 3; NC, negative control [Color figure can be viewed at wileyonlinelibrary.com]

To examine the role of KLF3 in lung cancer metastasis in vivo, 1 × 10^5^ A549 cells were intravenously injected into Balb/c mice. After 6 weeks, all mice were killed, and the lung tissues were histologically examined. As shown in Figure 6A and 6B, compared with mice injected with the control cells, mice injected with the shKLF3 cells had more metastatic nodules and larger metastatic nodules on the lung surface. In addition, shKLF3 A549 cells shortened the survival time of the injected mice compared with the shNC group (Figure 6C). Furthermore, the number of metastatic nodules, wet lung weight, and percentage of tumor tissues to total lung tissues in the shKLF3 group mice were much greater than those in the shNC group mice (Figure 6D‐F). To further evaluate the role of the KLF3/STAT3 signaling pathway in lung cancer metastasis in vivo, we performed a rescue experiment in a mouse model. As shown in Figure 6G‐I, compared with mice injected with the control cells, mice injected with the shKLF3 cells had more metastatic nodules and larger metastatic nodules on the lung surface; however, silencing STAT3 significantly abrogated the number and size of metastatic nodules induced by shKLF3. Overall, these results indicated that the KLF3/STAT3 signaling axis plays a crucial role in lung cancer metastasis.

### 3.7 Low KLF3 expression levels are associated with STAT3 expression in clinical lung cancer specimens

**Figure 7:**
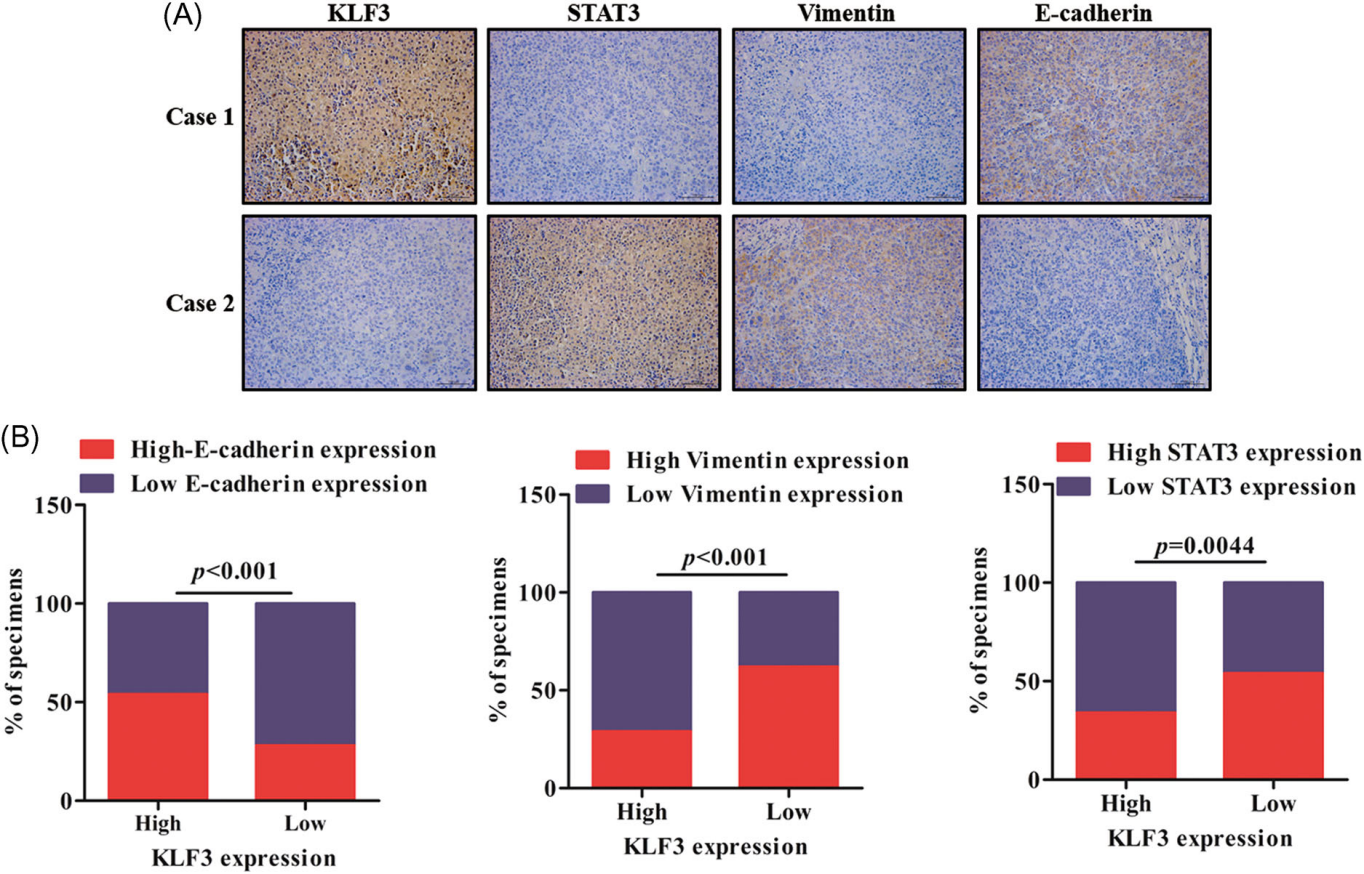
Low KLF3 expression levels are associated with STAT3 expression in clinical lung cancer specimens. A, Immunohistochemical staining of KLF3, vimentin, E‐cadherin, and STAT3 in human lung cancer specimens. Representative immunohistochemical staining images are shown. B, Spearman's correlation analysis between KLF3 expression and E‐cadherin, vimentin, and STAT3 expression in 56 cases of lung cancer specimens. KLF3, Krüppel‐like factor 3; STAT3, signal transducer and activator of transcription 3 [Color figure can be viewed at wileyonlinelibrary.com]

To understand whether KLF3 expression is related to EMT and the STAT3 signaling pathway in patients with lung cancer, we conducted an IHC assay to examine the correlation between KLF3, EMT marker, and STAT3 expression in 56 lung cancer specimens. As shown in Figure 7A and 7B, KLF3 expression was negatively correlated with STAT3 and vimentin expression in the clinical lung cancer specimens. Furthermore, there was a strong positive correlation between KLF3 and E‐cadherin expression. Overall, these findings strongly indicated that KLF3 acts as a crucial suppressor of lung cancer by regulating metastasis via STAT3 expression.

## 4 DISCUSSION

Metastasis is the major reason for a poor prognosis in patients with lung cancer. EMT is the key factor that causes epithelial cancer cells to lose cellular junctions and vessels to infiltrate distant areas.^24^, ^25^ To elucidate abnormal signaling pathways involved in metastasis could provide a potential therapeutic target for treating lung cancer. In this study, we demonstrated the molecular mechanism of metastasis regulated by KLF3 in vitro and in mouse models. Moreover, reduced KLF3 expression was found in lung cancer tissues, and KLF3‐mediated metastasis was shown to be dependent on the STAT3 signaling pathway. Therefore, therapies targeting KLF3 and STAT3 combined with current therapeutic strategies may act as an effective approach for treating metastatic lung cancer.

Recent reports have shown that KLF family factors play an important role in the development of tumors.^26^ For example, downregulated KLF expression has been observed in colon cancer. More significantly, reduced levels of this protein have been found in adenomas of patients with familial adenomatous polyposis, suggesting that the downregulated expression of KLFs in cancer seem to be a prognostic biomarker. In accordance with previous studies, we demonstrated that KLF3 expression is downregulated in lung cancer tissues and related to the TNM stage in this study. These results suggested that the expression levels of KLF3 are associated with lung cancer development and tumor metastasis. Increasing evidence has indicated that different KLF factors are involved in metastasis.^27^ For example, overexpressed KLF4 inhibits pancreatic cancer metastasis by regulating caveolin‐1 expression.^28^ Notably, Yan et al.^29^ also clearly showed that KLF8 promotes colon cancer tumorigenesis, invasion, and metastasis by transcriptional activation of FHL2. On the basis of reported observations, EMT is an important process in metastasis. Increasing evidence has shown that changes in cell phenotypes defined as an EMT play a role in the process of tumorigenesis, especially in metastasis.^30^, ^31^ Thus, we further examined the association between the expression levels of KLF3 and EMT markers, showing that the knockdown of KLF3 is accompanied by high levels of mesenchymal marker expression. In addition, we assessed the migration and invasion abilities of control cells and shKLF3 cells. Increased metastasis was observed in the shKLF3 cells compared with the control cells. To investigate the promotion of metastasis mediated by KLF3 knockdown, we performed animal experiments to validate the function of KLF3 knockdown in lung cancer. Consistent with the abovementioned data, the animal experiments showed that KLF3 knockdown markedly prompted lung cancer cells to localize to the lungs. Our data indicated that KLF3 is a key factor in lung cancer metastasis and that KLF3 mediates the induction of EMT to promote lung cancer.

Previous studies have shown that multiple signaling pathways are correlated with EMT changes and cancer metastasis. The IL6/STAT3 signaling pathway has been extensively explored in tumor progression. Importantly, increasing data have indicated that STAT3 activation is related to tumor metastasis and that STAT3 promotes lung cancer metastasis through EMT.^32^, ^33^ To investigate the precise molecular mechanism of regulation by KLF3 in lung cancer metastasis, we examined the potential abnormal signaling pathway involved in lung cancer that is mediated by KLF3 using bioinformatics analysis. Surprisingly, the STAT3 signaling pathway was enriched in downregulated KLF3 groups. In combination with previous reports and our bioinformatics analysis data, we speculated that KLF3‐mediated metastasis is dependent on the STAT3 signaling pathway. To validate this hypothesis, we examined the mRNA and protein expression levels of STAT3 in control cells and shKLF3 cells. Increased STAT3 expressions levels were observed in shKLF3 cells compared with control cells, indicating that KLF3 regulates STAT3 expression in a transcriptional manner. Previous reports have demonstrated that KLF3 exhibits repressive transcription activity by interacting with corepressors, such as mCtBP2 and FHL3.^34^ Consistent with these findings, we showed that KLF3 directly binds to the promoter of the STAT3 gene in lung cancer cells. Furthermore, we also found that the knockdown of KLF3 increased the luciferase activity of the STAT3 gene promoter but not the luciferase activity of the mutant promoter in lung cancer cells. Importantly, we performed rescue experiments to examine the role of STAT3 in metastasis mediated by KLF3 knockdown. The data showed that the prometastatic functions of KLF3 knockdown were attributed to the direct suppression of STAT3 expression, suggesting that reduced KLF3 expression constitutes a key mechanism that is responsible for lung cancer metastasis, complementing previously established molecular mechanisms of tumor metastasis.

Overall, our results indicated the crucial role of KLF3 in the regulation of lung tumor metastasis. Our data demonstrated that the knockdown‐mediated promotion of tumor metastasis is dependent on the STAT3 signaling pathway and that KLF3 expression is markedly related to lung cancer development in a clinical database. More importantly, we provided new insights into the KLF3/STAT3 signaling pathway in lung cancer. It has been reported that low expression levels of KLF3 in tumors are caused by DNA methylation.^16^ More importantly, DNA methylation inhibitors have been applied in clinical practice, suggesting that the expression of KLF3 might be promoted by DNA methylation inhibitors in clinical patients with lung cancer to improve the prognosis. Thus, we propose that KLF3 may be a poor diagnostic marker and a potential therapeutic approach for patients with metastatic lung cancer.

## CONFLICT OF INTERESTS

The authors declare that there are no conflict of interests.

## AUTHOR CONTRIBUTIONS

YD designed the experiments. WS and YD collected the clinical data and samples. WS and SH performed the experiments. ZY performed the statistical analysis. YD and ZY wrote and edited the manuscript. YD supervised the study. All authors have read and approved the final version of the manuscript.

## Supporting information

Supporting information
