# Supplementary material for: KLF3 is a crucial regulator of metastasis by controlling STAT3 expression in lung cancer
Source: Mol Carcinog. 2019 Sep 5;58(11):1933–45. doi: 10.1002/mc.23072 (PMC6852579; doi:10.1002/mc.23072)
Supplement: Supplementary file 1 — Supporting information [file MC-58-1933-s001.pdf]

Supplement Figure

A

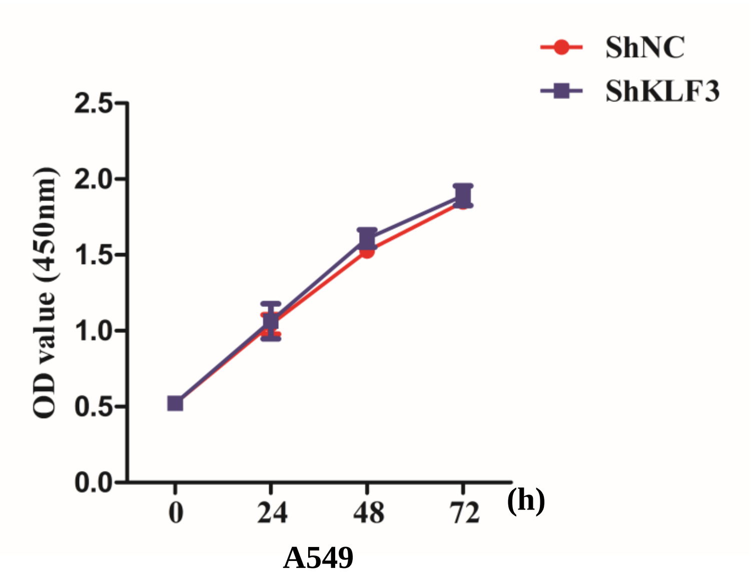

B

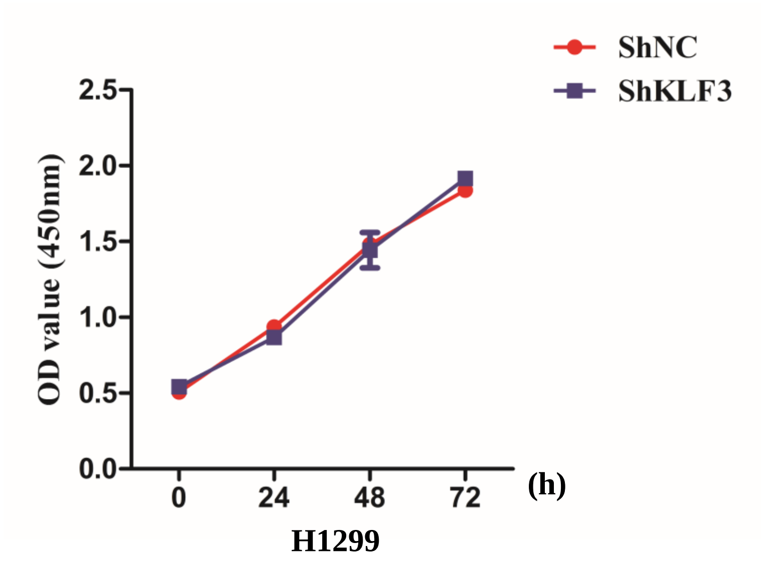

(A, B) CCK results showed the proliferative ability of ShNC cells or ShKLF3 cells both in A549 and H1299.
